# Five-Factor Personality Inventories Have a Competence-Related Higher-Order Factor Due to Item Phrasing

**DOI:** 10.3389/fpsyg.2020.557544

**Published:** 2020-11-26

**Authors:** Martin Bäckström, Fredrik Björklund, Rebecka Persson, Ariela Costa

**Affiliations:** ^1^Department of Psychology, Lund University, Lund, Sweden; ^2^Department of Psychology, São Francisco University, Campinas, Brazil

**Keywords:** personality, competence, traits, Big Five, higher-order factors

## Abstract

This research examines whether the items of some of the most well-established five-factor inventories refer to competence. Results reveal that both experts and laymen can distinguish between items that refer to how competently a behavior is performed and items that do not (Study 1). Responses to items that refer to competence create a higher-order factor in the personality inventories (Study 2), and the variability in responses to competence-related items in personality self-ratings is best modeled as a general factor rather than as also tied to the specific Big Five factors (Studies 3 and 4). We suggest that a focused debate on what personality items should refer to is likely to have considerable positive consequences for both theory and measurement of personality.

## Introduction

The present research concerns the concept of personality traits and how it has been defined and measured. The goal is to clarify the relationship between personality traits and the overall concept of personality. More specifically, we aim to show that trait theories and measures do not conform to the simple definition of traits as something related to individual differences in the frequency of behaviors, thoughts, and feelings, but instead, they also include other aspects, such as how well the behavior is performed, suggesting a particular level of competency.

More general personality theories often also include concepts that suggest such differences between people’s behaviors, as do theories of attachment, temperament, and agency and communion, just to give some examples. However, the present research will concentrate on the competence dimension within personality trait research. Trait theories differ in many ways, but the focus in the following will be on how both theoretical models and measures of personality traits fail to distinguish between the frequency of behaviors, thoughts, and feelings and how competent a person is in relation to a trait. Competence is a set of behaviors that provides successful outcomes (Bartram, 2005). In other words, competence concerns how well someone performs to achieve a goal. Our studies will investigate whether trait inventories have a competence-related dimension that is separable from the Big Five. Evidence of this dimension in ordinary measures of the Big Five would be important news, calling for a focused discussion on the implications so as to avoid inaccurate conclusions from studies in which the measures are used.

### Personality Traits, Competence, and Individual Differences

There have been many attempts to define personality traits, and several seem to concentrate on differences in behavior, thoughts, and feelings. For example, Funder (2001) suggested that personality traits are “an individual’s characteristic patterns of thought, emotion, and behavior” (p. 2). Although this way of conceptualizing personality is common, from a broad perspective, personality has also sometimes been almost equated to individual differences in general. This implies that if, for example, intelligence is defined as part of personality (e.g., DeYoung et al., 2014), competence is clearly included in the concept of personality. Within the field of individual differences, there are many concepts that do not focus on the frequency of behavior, thoughts, or feelings: cognitive abilities, knowledge, skills, emotional intelligence, creativity, and core self-evaluation. However, the purpose of the present research is not to suggest what concepts should be included in personality psychology but rather to investigate the consequences of mixing the quantitative aspect (frequency of behaviors) with what could be called the qualitative aspect (how well behaviors are performed).

### How Should the Relationship Between Competence and Personality Be Understood?

An important difference between personality traits and other traits, including competence, is whether they have been defined as unipolar or bipolar (Paunonen and Hong, 2015). A unipolar trait, like intelligence, has one low side (low intelligence) and one high side (high intelligence), while a bipolar trait rather has different behavioral tendencies at each pole, like introversion and extraversion. Paunonen and Hong (2015) have suggested that personality traits have two poles that represent approach vs. avoidance manifestations of a given trait. The average is thus a neutral point on a personality trait scale. With a unipolar trait scale, like in the case of competence, a very low score indicates lack of the given content. A low score on a personality trait scale instead indicates more than average approach or avoidance behaviors of the trait at hand. Paunonen and Hong (2015) have made a strong statement about polarity of traits, as they suggest that personality traits are not competence related and claim that unipolar traits should not be considered to be personality traits. Unipolar traits, such as skills, competencies, aptitudes, and intelligence, have no neutral point on which approach equals avoidance and a zero score would be considered a low end almost indicating an absence of the trait. This clearly suggests that at least some trait theoreticians do not think of traits as something related to competence. Saucier and Srivastava (2015) have suggested that already G. W. Allport pointed out that “…the more evaluative (or censorial) the term, the less reference to personality and the less value for the psychologist” (p. 283).

In summary, some theorists have claimed that personality traits are *not* unipolar, and if a trait is unipolar, it is not a personality trait. However, this view seems not to be representative of the general field of trait psychology. In the present research, we aim to contribute to a better understanding of the relation between trait models and measures used to estimate traits. In addition, we aim to add, by some novel empirical studies, to the knowledge of the consequences of an apparent lack of clarity between aspects that refer to the ability to perform a certain behavior (i.e., competence) and aspects that do not. What follows is a short review of some contributions to personality theory in which this distinction is made relatively clear.

### Personality Theories That Refer Directly to Competence

Many personality models refer quite directly to competence. For example, adult attachment theories employ concepts, e.g., secure or insecure variants of attachment styles, differentiating between persons depending on how well they adapt in romantic relations (Shaver and Mikulincer, 2012). Similarly, there are models of interpersonal traits (Jensen-Campbell et al., 2015) that explicitly suggest that traits influence how successful people are at getting along with others, to what extent they influence others and their vulnerability to interpersonal difficulties. Another example is the Leary and Toner (2015) theory of self-processes in the construction and maintenance of personality, which suggests that differences in self-concepts involve a distinct cognitive ability to think about oneself in the past and future, including feelings, thoughts, and motives, and the capacity to conceptualize and evaluate one’s characteristics, abilities, and actions. These theories, together with personality theories related to psychopathology (Markon et al., 2005), indicate that many personality psychologists have tried to tackle questions about adaptation and differentiated between traits that are more or less adaptive. In other words, they have suggested that some traits benefit persons overall, whereas others provide disadvantages. Another way of expressing this is that many researchers suggest that personality theory is about differences in the competency to adjust to the environment. There is a rather large gap between this perspective of personality traits and one that downplays the adaptive aspect, such as, e.g., Paunonen and Hong (2015).

### What Role Does Competence Have in Trait Theories?

The review above implies that differences in competence have not been properly distinguished from differences in the frequency of behaviors. Within personality theory, this has influenced trait models, and several concepts have been proposed to account for the differences in quality of traits. In particular, higher-order traits that are related to competence have been derived from analyses of empirical data. For example, it has been noted that traits tend to be organized at higher levels, with very general traits at the top (Goldberg, 1990, 2006). Goldberg described the very top trait as encompassing a general evaluation of the person, from good to bad, obviously a concept related to quality.

Another theoretical model related to the competence aspect of personality is the theory about the General Factor of Personality (GFP). Proponents of the GFP (see Musek, 2017) have argued that the Big Five personality factors all correlate and that the best explanation for this is a common content factor (Rushton et al., 2008; van der Linden et al., 2010; Musek, 2017). The GFP has been suggested to be strongly related to competence, for example, Musek (2017) states that “The GFP is a measure of general personal and social adjustment and can be defined as a dimension meaning high vs. low emotional stability, extraversion, conscientiousness, agreeableness, and intellectual openness” (p. ix). Rushton et al. (2008) suggested that the GFP represents traits that are conducive to successful reproduction and thus selected together over evolutionary time. Advocates of the GFP suggest that the GFP is related to social effectiveness (van der Linden et al., 2010). If inventories that measure the Big Five factors include many references to competence and social effectiveness, it is possible that this common content in the inventories is the reason why a general factor usually appears. We will pursue this possibility in the present research.

Others have suggested that the common content of personality should be organized as pairs of higher-order factors. Digman (1997) suggested two factors called the alpha and the beta to describe the higher-order content of the Five-Factor Model (McCrae and John, 1992). In the model, the alpha factor (agreeableness, conscientiousness, and emotional stability) is described as the socialization factor, and the beta factor (extraversion and openness) is described in terms of personal growth vs. personal constriction (Digman, 1997). The descriptions of the alpha and beta higher-order factors do imply that they include competence but to a different degree. The alpha factor could be interpreted as competence due to its relation to the individual differences in personality development. Note, however, that Digman’s version of the beta factor seems to be less of a unipolar higher-order trait since it includes both growth and constriction. The beta factor is somewhat more congruent with the Paunonen and Hong (2015) view of a proper personality trait. The alpha factor, on the other hand, could potentially be interpreted as a unipolar trait. This seems to suggest that the two higher-order factors are different regarding unipolarity and that the alpha factor is more obviously related to competence, compared to the beta factor.

DeYoung (2010) suggested a theory of the higher-order content of the FFM that resembles Digman’s alpha and beta factors. DeYoung (2010) has called his corresponding constructs “Stability” and “Plasticity.” The Stability higher-order factor (conscientiousness, agreeableness, and low neuroticism) has been described as both an ability and a tendency of individuals to avoid impulsivity and disruption and instead pursue goal-directed behaviors. Stability contains the same traits as Digman’s alpha factor and must be considered unipolar. The Plasticity higher-order factor (extraversion and openness/intellect) is described as the exploratory tendency in individuals that has them engage in the environment and to experience rewards (DeYoung, 2010). In other words, in DeYoung’s version, both higher-order factors are clearly defined as unipolar.

The quantitative conceptualization of personality traits presumes that someone who is extraverted performs more extraverted acts. This way of defining a trait has been extensively discussed and researched by Buss and Craik (Buss and Craik, 1983). They suggested that the relative incidence of acts within circumscribed categories or domains is what a trait is about. Using their example, “…the statement ‘Mary is arrogant’ means that, over a period of observation, she has displayed a high frequency of arrogant acts, relative to a norm for that category of acts” (Buss and Craik, 1983, p. 106), it is obvious that they deal with frequency of behaviors. Buss and Craik summarized their frequency approach to personality traits, suggesting that, for a given disposition, an act trend, or composite multiple-act index, is the basis for prediction of behavior. In Botwin and Buss (1989), the act frequency approach was compared to Big Five inventories, and when controlling for a general factor of number of acts, good correspondence was found. With regard to the present focus, it is interesting to note that many of the acts they suggested in fact included a reference to competence, “He paid his bills on time” and “He displayed knowledge of a foreign culture” are just two examples.

The above brief review of personality theories indicates that they have not taken seriously the distinction between how competently a behavior is performed and other aspects of individual differences. One of the many possible reasons for this is that some behaviors are considered clearly competent in one situation, e.g., talking to strangers in a selling situation, while the same behavior would have been deemed incompetent in another setting, e.g., during a funeral ceremony or a lecture. In other words, it is easy to forget that most behaviors can be competent in some contexts while less competent in others. This idea is very different from the idea that some people are, in general, more competent than others. For example, a unipolar concept like intelligence is clearly competence related, this is a concept that is uncomplicated to use in many situations, e.g., in recruitment and selection. In the same field, it has often been said that personality should be evaluated based on the content of the job (Tippins et al., 2018), clearly suggesting that personality traits’ relation to competence depends on the situation.

### Do Personality Traits Relate to Competence Criteria?

The perhaps most articulated separation of personality from competence is the distinction that has been made between personality and intelligence (Zeidner et al., 2001). However, not all personality psychologists accept this distinction. Cattell included intelligence as a factor in his 16-factor personality model (Cattell and Cattell, 1995). Furthermore, some modern trait psychologists argue that the Openness factor of the FFM is related to intelligence (DeYoung et al., 2014), as the motivation for cognitive exploration is strongly related to cognitive ability, i.e., competence. Similar ideas are present in the work of Allport, who saw intelligence as a factor of personality, because it “determines the quality and success of so many of the general adjustments of the individual” (Allport, 1921, p. 11).

Aside from whether personality traits and intelligence should be considered separate or not, some personality traits from the FFM have been shown to predict intelligence test performance (Chamorro-Premuzic and Furnham, 2004). Openness seems to be related to intelligence itself (Ackerman and Heggestad, 1997) rather than to intelligence test-taking like neuroticism (Furnham, 2001) and extraversion (Furnham et al., 1998). Therefore, it has been suggested that openness can be applied as a self-rated intelligence measure (Ackerman and Goff, 1994). Just like objectively measured intelligence, self-rated intelligence is a unipolar trait, and the correlations with openness and crystallized intelligence (Ackerman and Heggestad, 1997) could reflect a competence variance overlap.

Some theoreticians have a very strong position regarding what specific competencies are related to a certain trait. For example, Jensen-Campbell et al. (2015) argue for the specific competence that can be ascribed to a person with a trait, e.g., “Extroverted people compared with their more introverted counterparts have better social skills…” (p. 353), followed by “Extroverted people decode non-verbal behavior more effectively in situations that require attending to multiple tasks in the in environment than do introverts” (p. 353). On the other hand, Matthews (1999), in his review of the relation between personality and skill, a concept very similar to competence, suggests that this relation is more specific to certain situations that make adaptation easier for people with a certain trait. In addition, Matthews emphasizes that both sides of a trait, either extraversion or introversion, either emotional stability or neuroticism, can have adaptive advantages for a person.

Consiglio et al. (2013) have suggested the FFM as a theoretical frame of generic work behavior. In the study, they first constructed an inventory aimed at measuring FFM-related competences. They then, in an applied setting, investigated and compared self- and peer-ratings of FFM and their inventory to validate their competence model. They identified six competencies factors related to one or more of the factors from the FFM; Accomplishment and Process Management were mainly associated with Conscientiousness, Proactivity was mainly associated with Extroversion, Emotion management was mainly associated with Emotion Stability, Teamwork was mainly associated with Agreeableness, and Innovation was mainly associated with Openness.

### Do Big Five Models Refer to Competence?

It seems fair to say that many personality theories include concepts related to quality and not only quantity. But even within the Big Five model, traits vary in how strongly they are associated with competence. For example, the introversion–extraversion factor is not expressively concerned with the competence of the behavior. Behaviors such as seeking danger, laughing out loud, visiting friends, and so on do not necessarily imply a certain competence level. Conversely, however, the conscientiousness dimension of the same model often seems to denote competence. The Big Five model of McCrae et al. (2005) even includes competence as a subfactor of conscientiousness.

### Measurement of Personality Traits and Hypotheses

Trait theory has been almost as dependent on ratings as the cognitive theories of intelligence have been dependent on the results from intelligence tests. This is evident in the dependence between the lexical hypothesis and many trait models. According to the lexical hypothesis, important differences between people have been coded in the language. This makes the content of what words are included in the lexical analyses extremely important for the theory that has been built on it. For example, by analyzing how people rate themselves and others on a large number of selected words (Goldberg, 1990), personality theorists could replicate the most popular model within trait psychology, i.e., the Big Five model. Before the lexical trait models were developed, there were, however, several researchers who worked with categorizing words related to individual differences (Allport and Odbert, 1936; Norman, 1967). For example, Angleitner et al. (1990) categorized such words and found six important sets of categories relevant for differences between people, but only the first, traits and abilities, has been employed to select personality content (Saucier, 2019). For the present work, it is important to note that the subcategory “abilities” was one of the most frequent in both English and German. The words chosen in the lexical analyses have been the base material for making many personality inventories, and the first category, containing traits and abilities, has been the major category to select words from.

One possible consequence of selecting words from both “traits” and “abilities” is that the factors of trait models will include both ability factors and trait factors. As was reviewed above, some traits seem to be more closely related to abilities than other traits, e.g., conscientiousness. Other traits can also include abilities, for example having the ability to be friendly, sympathetic, open-minded, and keeping calm. This is directly related to the main question asked in the present research, which is whether personality inventories refer to both quantitative aspects (e.g., behaviors like spending time with friends) and aspects referring to abilities and competencies. If so, this could indicate that traits, which often have been defined by their measures, are a combination of both. Our general hypothesis is that many inventories include scales that mix items that refer to competency aspects with items that do not. In the empirical studies of the present research, we will first test whether there is competence variability in personality inventories and investigate whether it is possible for experts and laymen to distinguish between competence-related items and items that are less related to competence. After investigating this, we turn to the consequences of such a mix. More specifically, we test the hypothesis that items that are competence related will influence raters and result in factors, either general or more specific, that are relatively independent of the personality factors. We will test two different possibilities: (1) that competence forms a single higher-order factor and (2) that competence-related items form separate factors, after controlling for the single higher-order factor, specific to each factor. In total, four different studies will investigate this, based on different measures and samples.

## Study 1

Theoretically, items in personality inventories, based on trait theories, are designed to measure quantitative individual differences in behaviors, thoughts, and feelings (Matthews et al., 2003). The question investigated in this study is whether personality inventories (e.g., NEO-PI-R) also include items referring to competence-related differences in thoughts, feelings, and behaviors. That is, to what extent personality inventories mix items referring to the frequency of thoughts, feelings, and behaviors and the competence with which thoughts, feelings, and behaviors are performed.

The first study will investigate whether experts and laypersons can differentiate between items with a competence content and items with less connection to competence. We expect to find that both experts and laypersons can recognize competence content in items and that there is a mix of both competency and frequency content in items of standard personality inventories. We focus on the amount of such items in relation to items that do not refer to competence. Examples of competence-related items are “Get chores done right away” from the IPIP-300 inventory (Goldberg et al., 2006), “I never seem to be able to get organized” from the NEO-PI-R (Costa and McCrae, 2008), and “Stays optimistic after experiencing a setback” from the BFI2 (Soto and John, 2017). All of these have a content that refers to competence. The first item describes a person who is capable of quickly finishing tasks, whereas the second rather describes the opposite. The third item describes a person who has the ability to adapt to negative events. These items can be compared with, “Love large parties” from the IPIP-300, “Sometimes I bubble with happiness” from the NEO-PI-R, and “Feels little sympathy for others” from the BFI2, items that are much less competence related, called “non-competence items.” The first of these describes an attitude, the second an emotional expression, and the third (low levels of) an emotional state. Having these attitudes or emotions does not imply competence. Our research question is not whether there exist items that depict competence, but to what extent some popular personality inventories contain such items and whether people agree regarding which items should be categorized as competence related.

### Materials and Methods

#### Participants

Two types of raters participated in Study 1: expert raters and layperson raters. There were four expert raters (the authors), all of whom are familiar with trait theories and the Big Five model. The laypersons were students recruited through the Prolific platform. Four different inventories were rated by 30 participants per study. The distribution of male and female participants and their mean age are displayed in Table 1. The inclusion criteria were the following: participants were to be 18–30^1^ years old, have origin in a country with English as its main language, to not have participated in any of our previous studies, and be students.

**TABLE 1:** Sex and age of the competency categorization samples of Studies 1 and 3.

Inventory	No reported sex	Women	Men	Age mean	Age *SD*
NEO PI-R	0	13	17	23.23	5.46
BFI2	3	13	14	23.41	5.13
IPIP300	1	21	10	26.77	6.73
BCBI	0	21	9	23.13	5.75

*BCBI, the Behavior and Competence-Based Big Five.*

#### Measures

Two of the inventories, the BFI2 and the NEO-PI-R, were included because they are among the most well-known and cited inventories measuring the FFM/Big Five model. A third inventory, the IPIP-300, was included since very large samples of data from it are openly accessible. The fourth inventory was created to be used in Study 3 and will only be briefly presented here.

The NEO-PI-R (Costa and McCrae, 2008) is a measure of the Big Five personality domains, namely, Neuroticism (N), Extraversion (E), Openness to experience (O), Agreeableness (A), and Conscientiousness (C). Participants respond on a 5-point rating scale. The inventory is one of the oldest and most well-established five-factor personality inventories available. It has shown validity in many areas, both theoretical and applied. The inventory consists of 240 items, 48 items for each domain.

The BFI2 (Soto and John, 2017) is a new version of an inventory by John et al. (1991). It was developed to measure the prototypical features of each Big Five domain. The new version has more items (60 instead of 44) and better psychometric properties. The items are rated on a 5-point scale ranging from disagree strongly to agree strongly. The domain scale homogeneity, as measured by Cronbach’s alpha, has been above 0.80 in all domains. It is freely available for researchers to use; it is comparatively short and has shown high validities.

The IPIP-300 is a Big Five inventory from the International Personality Item Pool (Goldberg et al., 2006) that is free to use and has shown congruent validity in relation to the NEO-PI-R. The inventory has 300 items distributed over the five Big 5 factors, which have six facets each. The IPIP-300 has been used in a number of studies and has shown high factor scale homogeneity (above 0.80) for all factor scales.

The last inventory, called the Behavior and Competence-Based Big Five (BCBI) inventory, is also based on the item set from the IPIP. However, the 200 items that represent the five factors are, by design, either behavior or competence related. For each item that was behavior related, we created a new item as similar to the original as possible but with a clearly more competence-related content and vice versa. The final inventory had 100 behavior-related items and 100 competence-related ones^2^.

#### Procedure

The experts made their ratings starting with the IPIP-300, followed by the NEO-PI-R, BFI2, and last the BCBI. The expert ratings aimed to investigate whether it was possible to reach a high level of agreement on which items contained reference to competence. This was needed to get a clear-cut instruction that would later be provided to the laypersons. The laypersons categorized items in a web application that presented items one at the time, in a unique random order for each participant. The instruction for the laypersons used the following definition of competence: “Your task is to read a number of items and determine whether they include a description related to how competent a person is. Items describe competence if the content refers to having (categorized as 1) or lacking (categorized as 0) the competence to succeed with something. For example: “Am efficient,” “Have difficulties reading some texts,” “Anticipate others’ needs,” “Cannot control my irritability,” but also “Make friends easily” all include a description of how competent the person is. Remember, also items describing an incompetent person concern how competent the person is.”

To estimate the amount of agreement within the expert and the layperson group, we used two types of intraclass correlations (ICC). The single ICC estimates the mean agreement for any single rater, and the average ICC estimates the agreement (reliability) for all raters taken together (very similar to Cronbach’s alpha). Analyses was conducted in R (R Core Team, 2019) also using the psych package (Revelle, 2017).

### Results

The first study investigates whether personality inventories contain items that are competence related, distinguishable from items that are not competence related. Ahead of the first ratings by the experts, the categorization of items was tested in a pilot study that included 30 Swedish students. In this study, a preliminary version of the instruction was used, and the task was to categorize items as referring to either competence, behavior, or neither. The results of the pilot study indicated that it was possible to distinguish competence items from other items.

The experts rated the items starting with the IPIP-300 inventory. The goal of the expert ratings was twofold: first to investigate whether there was agreement on which items should be categorized as competence related and second to try to increase the clarity of the instruction. As instruction for the task, we used the same definition of competence as in the pilot study. The individual ICC, i.e., which is the estimated agreement between all pairs of raters (Shrout and Fleiss, 1979), was 0.54, which indicates some agreement among raters. The average ICC was as large as 0.87, indicating that, overall, the four experts agreed on what items could be categorized as competence related. There was, however, a rather large difference between individual raters, for example the number of competence-related items varied, from a proportion of 0.29–0.48. The individual ICC was 0.54 and 0.39, and the average ICC was 0.84 and 0.72 for the NEO-PI-R and the BFI2 inventories, respectively. Generally, even if the instruction was somewhat adjusted between rounds, the agreement did not increase. Laypersons were asked to categorize the items as either being competence related or not. The most common competence-related items from each of the three inventories are displayed in Table 2, and results from the three inventories are displayed in Table 3 (together with a fourth inventory used in Study 3).

**TABLE 2:** The most often selected competence items from each factor of the three inventories.

Test	Item	Factor	Percent competence rated (%)
IPIP 300	Complete tasks successfully	Conscientiousness	100.00
	Adapt easily to new situations	Neuroticism	97.00
	Can handle complex problems	Openness	97.00
	Take charge	Extraversion	88.00
	Feel sympathy for those who are worse off than myself	Agreeableness	84.00
NEOPI-R	I try to do jobs carefully, so they won’t have to be done again.	Conscientiousness	97.00
	I feel I am capable of coping with most of my problems.	Neuroticism	93.00
	Once I find the right way to do something, I stick to it.	Openness	83.00
	Human need should always take priority over economic considerations.	Agreeableness	83.00
	My work is likely to be slow but steady.	Extraversion	80.00
BFI2	Is inventive, finds clever ways to do things	Open-mindedness	97.00
	Is efficient, gets things done	Conscientiousness	93.00
	Is dominant, acts as a leader	Extraversion	90.00
	Is relaxed, handles stress well	Negative Emotionality	83.00
	Is helpful and unselfish with others	Agreeableness	83.00

**TABLE 3:** Layperson and expert categorizations of personality inventory items.

	Laypersons (*N* = 30)			Experts (*N* = 4)		
Inventory	Individual measures ICC	Average measures ICC	Proportion competence items (%)	Individual measures ICC	Average measures ICC	Proportion competence items (%)
NEO PI-R	0.177	0.840	50.6	0.541	0.825	34.5
IPIP-300^*a*^	0.247^*a*^	0.913^*a*^	46.0^*a*^	0.524	0.815	31.8
BFI2	0.227	0.898	51.7	0.392	0.721	36.5
BCBI	0.224	0.897	48.1	×	×	×

*Proportion of competence items was defined as items with more than 50% rated them as competence related. ^*a*^N was 32 for this inventory.*

The two different ICCs for the laypersons (see Table 3) showed that they agreed (high average ICCs), but that this agreement was rather low when individual participants’ ratings were compared (rather low individual ICCs). Agreement was somewhat stronger in relation to the IPIP-NEO, but all inventories had impressive figures of average agreement.

Expert and laypersons ratings correlated moderately to high, *r* = 0.58, 0.57, and 0.72, for IPIP-NEO, NEO-PI-R, and BFI2, respectively (all *p*s < 0.001). Agreements were larger for ratings of non-competence to items from IPIP-NEO and NEO-PI-R, but laypersons also rated items as relatively higher in competence (1) compared to non-competence (0) both for the IPIP-NEO, 0.47 against 0.39, and the NEO-PI-R, 0.51 against 0.34. For the BFI2 items, ratings of items were more similar: laypersons, 0.52 and experts, 0.53. Table 3 also has the number of items that were rated as competent by more than 50% of the raters, both for experts and laypersons. To summarize, there were both similarities and differences in how items were rated.

While Study 1 shows that some items are competence related, it is somewhat puzzling why test constructors have mixed items in this manner. Whether including competence-related items is an intentional feature or something that has accidentally been included in Big Five inventories is a question for future research. Before discussing this, we will make some attempts to investigate whether ratings are influenced by the competence information in inventories. Study 2 will investigate whether items that have been rated as related to competence influence self-ratings of personality traits.

## Study 2

In Study 2, we will test the hypothesis that competence content in items creates a separate factor in personality self-ratings of typical Big Five inventories, using several data sets. This question is closely related to the hypothesis about a GFP, where it has been shown that many personality inventories have a separate higher-order factor (van der Linden et al., 2010). The information gathered about items in Study 1 will be used to create two different versions of the tested inventories, one based on competence items and the other on all other items. Note that in Study 1, about 50% of the items were categorized as competence items by the laypersons. This makes it possible to create two different versions of the inventories, one competence related based on half of the items and one non-competence related, based on the other half.

### Materials and Methods

#### Participants and Measures

Study 2 makes use of existing data sets of different origins (detailed information is provided in the specific publications). For the IPIP-300, we use a very large English sample from Johnson (2014). The IPIP-300 sample had 397,313 participants, of which 60.1% were female, and the mean age was 25.2 (*SD* = 10.0). For the NEO-PI-R, we used the Eugene-Springfield community sample (Goldberg, 2008). This sample had 1,124 participants, of which 53.1% were female, and the mean age was 49.7 (*SD* = 13.1). For the BFI2, we used a Prolific sample of 249 participants collected by ourselves, 53.0% were female, and the mean age was 24.9 (*SD* = 3.3). The Prolific sample consisted of students from either the United Kingdom or the United States. The Prolific participants made their ratings on a proprietary Internet site created just for this occasion; they registered with their anonymous Prolific code and were paid £3 for their participation^3^. For all three inventories, items were rated on a 5-point scale.

#### Procedure

Information from Study 1, where items were categorized as either competence related or not, was used in Study 2 to create new inventories that either included competence-related items or not. The two versions were based on either the categorization by experts or laypersons, resulting in four different inventories (see Table 3). We selected items that were categorized as competent vs. non-competent by at least 60% of the raters.

NEO-PI-R and IPIP-300 were analyzed on the facet level. Facets were created based on the items that were rated as either competence or not competence related. The included facets in the competence sets and the non-competence sets were not always the same, and some facets did not include items that could be classified into the two sets, e.g., the facet sympathy from agreeableness in the IPIP-300 did not contain any competence related items.

#### Statistical Analyses

To test the hypothesis that competence forms a separate factor, we used two different strategies. The first was based on principal component analysis (PCA) and the second on confirmatory factor analysis (CFA) as estimated with MPLUS 8.1 (Muthén and Muthen, 2017). Principal component analysis summarizes the amount of common variance in a number of variables, and the first component always represents the largest amount. A large first PCA supports a general factor when all or most of the variables have significant loadings to this component. In CFA, it is possible to test more directly how much variance can be accounted for by a common factor. One simple way to do so is to estimate a single factor with all loadings fixed to 1. To avoid making the estimations too dependent on unreliable observed variables, we excluded all facets that only had a single item. The normed fit index (NFI) is an estimate of the amount of covariance relative to the null model. Since the competence and the non-competence variants have a somewhat different number of observed variables, the CFI will be used; this fit index is similar to NFI but compensates for how many variables that are included in the model. In addition, we also estimated the hierarchical omega as implemented in the psych package (Revelle, 2017) using the R program environment. The hierarchical omega is an estimate of the general factor saturation of a test. In the present analyses, the omega is estimated based on exploratory factor analyses. To support that there was more common variance in the competence versions of the inventories, both the first PCA, the CFI, and the hierarchical omega should be larger for the competence versions. It is important to remember that the non-competence versions included somewhat more facets. The number of items in the BFI2 was, however, about the same. Note also that the facets were not the same. For the IPIP-300, for example, the competence versions had a total of 22 facets with more facets from the conscientiousness (6) and neuroticism factors (4). The non-competence version had 27 facets and included more facets from extraversion (6), agreeableness (6), and openness (6) but of course included conscientiousness and neuroticism facets as well.

### Results

Table 4 displays the results from the PCAs of both the competence and the non-competence versions of the inventories. It is obvious (see PCA1 columns in Table 4) that all three inventories have a larger first PC when only competence-related items are used. With the exception of the NEO-PI-R, the amount of variance accounted for was about the same for the analyses based on the expert and layperson ratings, but the difference between competence and non-competence was generally larger for the expert ratings. There is an important difference between the BFI and the other two inventories in that the analyses of BFI were based on the 60 items, while the other two inventories were analyzed on facet level.

**TABLE 4:** Strength of first principal component and first factor (CFA) for non-competence and competence inventories.

	Non-competence				Competence			
Inventory	PCA1 (%)	Facets/#items	CFI	CFI#1	PCA1 (%)	Facets/#items	CFI	CFI#1
**Experts**								
NEO PI-R	17	27/#152	0.32	0.08	28	19/#69	0.61	0.17
IPIP-300	20	27/#169	0.31	0.19	26	22/#94	0.47	0.14
BFI2	25	#20	0.42	0.38	34	#21	0.60	0.57
Laypersons								
NEO PI-R	17	23/#74	0.40	0.15	21	24/#87	0.56	0.14
IPIP-300	22	24/#131	0.39	0.15	26	23/#83	0.58	0.22
BFI2	27	#25	0.46	0.40	34	#24	0.63	0.58

*The analyses of NEO PI R and IPIP-300 uses subscales. BFI2 uses items. CFI, Comparative Fit index; CFI#1, Comparative Fit Index of the fixed model; PCA1, first principal component; #, number of items in the inventory*

Using CFA, we tested two different models. The first estimated all loadings, while the second put equality restrictions on the loadings. Table 4 shows that all models based on competence-related items had higher CFI; the difference from non-competence-related items was obvious. Since CFI can be interpreted as the proportion of covariations that is represented by the model, it is possible to conclude that the first factor represented almost half (≈44%) of the covariance. The results are not as clear cut for bifactors, to which there were equality restrictions. This suggest that the common variance was not equally distributed over all scales. This is hardly surprising since the number of competence items was not evenly distributed over all scales. Another possible interpretation of this is that inventories include more than one higher-order factor, like Digman’s alpha and beta factors.

To complement these analyses, we also estimated hierarchical omega. The results in Table 4 supported the other analyses for all inventories and groups except IPIP-300 for the laypersons. For omega, the inventory with the strongest hierarchical competence factor was BFI2.

The competence-loaded items/scales tended to create a larger common factor in the inventories, which could be a problem since it interferes with the simple structure of the personality model. Basically, the results suggest that scales tend to be even strongly correlated when competence has been included. On the other hand, our support for the validity of the competence factor rests only on content validity showed in Study 1. We will attempt to test the criterion validity of the factor in Study 4. It is also important to note that there was a general factor also in the non-competence versions. We did not make any formal significance test on the difference. This is not necessary since two of the samples were large and the third (the one used for the BFI) exhibited a very large difference between competence and non-competence.

It is a limitation of the strategy used in Study 2 that neither the number of scales/items nor the facets of the different inventories were the same (although most facets were included). This will be addressed in Study 3, which uses a different strategy. A new inventory will be introduced, designed to have the same number of competence and non-competence items in both versions.

## Study 3

This study will investigate the hypotheses suggesting that competence variability can be found in personality inventories as a higher-order factor and, in addition, that specific competence factors can be found related to each of the Big Five. We will attempt to test this by creating a new inventory that was deliberately constructed to estimate specific competence factors. Because it can be suspected, based on Study 2, that there will be a large general factor also in this inventory, we will investigate the specific competences defined as unique to both the Big Five factors and the general factor.

### Materials and Methods

#### Participants

There were two samples in this study. The first sample consisted of 30 Prolific users (see Table 1 for demographics). The second sample consisted of 200 Prolific users, 88 men and 112 women. Their mean age was 23.6 (*SD* = 5.03). For both samples, the inclusion criteria were that they came from an English-speaking country, that they were between 18 and 30 years old, that they were included in the Prolific definition of a student, and that they had not participated in a previous study in this project.

#### Measures

To investigate the hypotheses, we created two new inventories based on the IPIP-100 Big Five inventory (Goldberg et al., 2006). The goal was to have one inventory that consisted of items with clear competence content and one with minimized competence content.

First, the items of the IPIP-100 inventory were rated in relation to competence by all authors. Based on these ratings, we created two list of items. For each item that had been rated as competence related, we created a new item that was less competence related, and for each item that was not competence related, we created a competence-related version of the item. The goal was to create items that were similar in content (e.g., still measured the same facet of the factor) but dissimilar regarding competence. The items were rated by a small group of Prolific users (*N* = 30), and, as could be expected, it was found that the items differed regarding competence (see Table 3, items in Supplementary Material). In the second step, a second group of Prolific users made self-ratings on the items of both inventories, on a 5-point scale.

#### Procedure

The participants rated themselves on a five-step Likert scale on a proprietary Internet page^4^. The instruction was to rate according to how well the items fitted their usual behavior. The items were presented in a unique random order to each participant.

#### Statistical Analyses

In the first analysis, we will test whether the general factor based on competence scales is relatively larger than the factor based on non-competence scales (as was the case in Study 2). The second analysis tests whether there is unique variance in the general competence factor not included in the general factor based on non-competence items. The third analysis tests whether it is possible to identify unique competence factors for each of the five personality traits of the Big Five.

The hypotheses were tested with PCA and CFA using the psych package (Revelle, 2017) and MPLUS 8.4 (Muthén and Muthen, 2017), respectively. To estimate the competence and non-competence versions of the inventory, we created four random parcels from each factor of each inventory. In total, there were 40 observed variables, four competence and four non-competence variables from each factor. When testing the third hypothesis, we used even more aggregated variables, two competence and two non-competence parcels for each factor.

### Results

The first hypothesis was that the general factor was stronger for the competence inventory than for the non-competence one. This was first tested with PCA using the five-factor scales, and it was found that the first PC was larger in the competence-related inventory than in the non-competence related inventory. The first PC explained 45 and 37% variance, respectively. When estimating a PCA on the item level, the difference was 18% common variance compared to 15% in the non-competence-related inventory. The mean loadings were 0.40 (*SD* = 0.11) and 0.35 (*SD* = 0.15), for the competence and non-competence inventories, respectively. This difference in loadings was significant, *t*(188.42) = 2.63, *p* < 0.01. The results replicate the results from Study 2.

The second hypothesis concerned the strength of the relation between the general factors of the competence and the non-competence inventories. This was tested by CFA including both inventories. Two different models were tested. The first model was based on the hierarchical model. It included the two FFM structures and two general factors for competence and non-competence, respectively. The second model was a bifactor model, with five FFM factors and two general bifactors that were correlated. There were problems with estimating both models, as the latent variable covariance matrix was not positively definite, suggesting multicollinearity. This could be attributed to a very strong relation between the two general factors, i.e., the one from the competence-related inventory and the non-competence-related inventory. The standardized coefficient was larger than 1.0. Both the bivariate and the hierarchical model had this problem. Even if it was not possible to make a formal test of the relationship, the results suggest that the general factor from the competence and the non-competence inventories, while differing in size, were based on the same underlying factor. Given the strong correlation between the general factors, we also estimated the correlation between the first PC of each inventory, which was found to be 0.94. Since the components are not estimated without error (like the latent variables), this is a result that supports an almost 1.0 relation between the general factors.

The hierarchical model included the two different versions of the FFM-observed variables. In this model, it was found that all factor pairs, with one from the competent and one from the non-competent variants, were almost completely collinear, i.e., had a latent variable correlation of 1.0 (only the openness factor pair revealed a somewhat lower correlation, *r* = 0.94).

**FIGURE 1:**
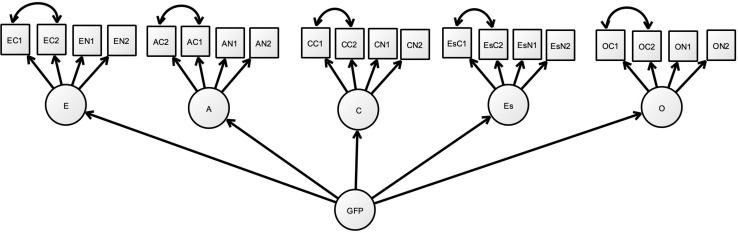
Model used to test for specific competence factors. Abbreviations first letter: E, Extraversion; A, Agreeableness; C, Conscientiousness; Es, Emotional stability; O, Openness. Second letter: C, Competence; N, Non-competence. Third letter: 1, parcel #1; 2, parcel#2; GFP, general factor of personality. Covariance arrows are the ones tested for specific competences.

The third hypothesis proposes that there are unique variances related to the specific competence factors. To test this hypothesis, we created a model with five factors for the FFM and one bifactor representing the common factor of all variables; this was the base model. Only four parcels were used as indicators for each of the FFM factors, two from the competence and two from non-competence inventory, respectively (see Figure 1). The model had rather good fit, χ^2^(150) = 286.3, root mean square error of approximation (RMSEA) = 0.067; comparative fit index (CFI) = 0.971. We then added the correlations between the residuals for the competence variable pairs. The change in model fit was not significantly better, χ^2^(145) = 277.1, delta χ^2^(5) = 9.2, *p* > 0.05, RMSEA = 0.067, CFI = 0.972. Among the five correlations tested, one for each factor, only the correlation between the openness residuals was significant (*p* < 0.02). In other words, the results did not support possible separate competence factors. Another test of the same hypothesis was based on eight indicators per factor, four from each inventory. In this case, we tested whether there was support for separate competence latent variables, but as above, again, the only factor with a significant latent factor was the openness factor^5^; the support was very weak for specific factors, in other words, the hypothesis tested was not supported.

In summary, the competence inventory seemed to have a somewhat stronger general factor. This factor was, however, based on the same variance as the general factor of the non-competence inventory. This suggests that they both capture the same content, only that this content was more prominent in the competence inventory. There was little support for attributing variability in the competence inventory to the specific Big Five factors. The only factor for which some support was revealed was the openness factor.

Studies 1 and 2 showed that it is possible to differentiate between personality items that are competence related and not. However, in Study 3, it is not so clear how the items, being competence related or not, affected the self-ratings. It appears that including competence-related items activates evaluative responses for some participant and that this in turn increases the correlation between items. This line of thought was supported by the 1.0 correlation between the higher-order factors. In addition, including competence items did not create specific competence-related information unique to the five factors. There was little support for specific competence factors (i.e., unique errors of competence items or latent specific competence variables). This is of course a null result and should be interpreted with caution.

## Study 4

The last study will test a somewhat different, but related, hypothesis. Many personality trait models seem to include a competence dimension or even include explicit competence concepts (e.g., the competence facet of NEO-PI-R). There have even been attempts to create a Big Five competence model, resting on the assumption that competence is organized around the Big Five traits, i.e., there are specific competences related to extraversion, other competences related to openness, and so on. For example, Consiglio et al. (2013) have created a work-competence model based on the Big Five. In this model, there are six factors that map on the personality factors: proactivity on extraversion, accomplishment and process management on conscientiousness, teamwork on agreeableness, emotion management on emotional stability, and innovation on openness. They found support for specific relations between the personality traits and work competencies that matched their mapping. To test their model, they used a newly created inventory that operationalized the six competence factors, the BFC-GRID. They found support for a very general competence factor and also that the general factors based on self- and peer ratings had a rather weak correlation (about 10% common variance). The last result is in line with other studies showing a weak relation between self- and peer-rated GFP (e.g., Anusic et al., 2009; Danay and Ziegler, 2011; Bäckström and Björklund, 2014).

Studies 1–3 in the present research supported a competence factor in Big Five inventories, but in Study 4, returning to the third hypothesis of Study 3, we investigate whether a dedicated competence model makes it possible to reveal unique Big Five competencies, or alternatively whether the competence factor from a Big Five inventory explains the competence variability of the dedicated competence model.

### Materials and Methods

#### Participants

The present study used Prolific workers, from countries with English as their mother tongue; 101 were students, and 100 were from a mixed group of non-students. In the last group, we only included subjects with full-time jobs (according to their own reports to Prolific). The sample included 93 male and 108 female subjects, and their mean age was 26.9 (*SD* = 6.35).

#### Measures

To measure the Big Five traits, we used the IPIP-300 (see Studies 1 and 2) but only included 20 facets, 4 from each factor. This inventory contains 200 items, 10 for each facet. The facets are the following: Extraversion—Active, Friendliness, Excitement Seeking, and Happy; Agreeableness—Altruism, Morality, Sympathy, and Cooperative; Conscientiousness—Achievement Striving, Dutifulness, Self-Efficacy, and Orderliness; Neuroticism—Vulnerability, Anxiety, Anger, and Depression; Openness—Emotionality, Intellect, Adventurousness, and Artistic.

The BFC-GRID (Consiglio et al., 2013) is an inventory that measures work competencies organized according to the Big Five. It encompasses 40 items. The inventory has six scales measuring Process management (Big Five C, six items, α = 0.69), Proactivity (Big Five C E, six items, α = 0.75), Emotion management (Big Five Es, eight items, α = 0.69), Teamwork (Big Five A, eight items, α = 0.83), Innovation (Big Five O, seven items, α = 0.79), and Accomplishment (Big Five C, five items, α = 0.65). The inventory was psychometrically investigated by Consiglio et al. (2013), who found support for reliability, criterion validity, and construct validity, both with self- and peer-ratings. We used the same response format as Consiglio et al. (2013) did, a 7-point Likert scale anchored with Never and Always.

#### Procedure

The participants were invited to take part in the study through the Prolific service. They were referred to a proprietary web application where they were instructed and where they performed their ratings. The personality inventory was administered first, followed by the work competence scale. Between the two inventories, a short instruction was given to alert the participants about the response format of the BFC-GRID.

#### Statistical Analyses

The main hypothesis in this study will be tested by estimating CFA models. Before testing this hypothesis, we will again test the existence of a general factor to replicate what was found in Consiglio et al. (2013). Each subfactor of the BFC-GRID was estimated by three randomly aggregated parcels based on two to three items. Two items of the emotion management scale were not related to the other items in the scale in the present study and were therefore deleted before parceling. The Big Five factors were estimated using the facet scales (four to each factor). We tested the hypothesis that there are unique competence factors in the BFC-GRID when the variance from general factors of both the BFC-GRID and the Big Five instrument, and in addition the Big Five factors, have been accounted for. This model is a MIMIC^6^ model that tests a CFA model with covariates. We used the MPLUS 8.4 to estimate all models (Muthén and Muthen, 2017). In our model, the factors created for the BFC-GRID has one covariate from each designated Big Five factor, plus the general factor from the Big Five. The model is depicted in Figure 1. The hypothesis regarding the unique specific competence factors will be tested by investigating the residuals, i.e., the left-out variance of the factors, after adding the general factor and then the personality factors as covariates. The residuals will include all variance that is unique to each factor, which is possible because the factors estimated in CFA are latent, i.e., do not contain error variance.

### Results

First, the measurement models of the BFC-GRID and the IPIP-300 inventory were tested. The BFC-GRID measurement model, with one general factor, had good fit, as suggested by all three fit indexes, χ^2^(130 = 245.0, RMSEA = 0.066, standardized root mean square residual (SRMR) = 0.052, and CFI = 0.93. The loadings on the latent variables were all above 0.59 and below 0.85, with a mean of 0.71. We first investigated whether there was a general factor in the BFC-GRID. We tested this hypothesis, or the significance of the general factor, by setting all loadings of the general factor to 0 (also the covariance of the general factor was set to 0). This model had much worse fit, χ^2^(135) = 906.2, RMSEA = 0.169, SRMR = 0.342, and CFI = 0.528, the difference in χ^2^(5) = 661.2, *p* < 0.001.

The specific competence factors had very strong loadings on the general factor, indeed so strong that the residual of the accomplishment factor was negative (this negative residual was fixed to 0 already when the measurement model was tested). This suggests that there was no specific variance in the accomplishment factor; all could be attributed to the general factor. In addition, two residuals, the ones of the process factor (res. var. = 0.12) and the proactive factor (res. var. = 0.10), were non-significant (*p* > 0.05), indicating that the general factor explained most of the variance also in these two competence factors.

The fit of the measurement model for the IPIP-300, with one GFP, was very bad; all fit indices indicated bad fit, χ^2^(165) = 1,018.7, RMSEA = 0.160, SRMR = 0.138, and CFI = 0.643. We decided to create an additional measurement model with modifications and use this model to control whether the covariance not accounted for in the first model changed the results. The model with added secondary loadings and error correlations had 29 less degrees of freedom, and fit indices suggested adequate fit, χ^2^(136) = 280.3, RMSEA = 0.073, SRMR = 0.059, and CFI = 0.940.

We investigated whether a general factor based on the BFC-GRID correlated strongly with the general factor of the personality inventory. We merged the competence model and the Big Five model (without modifications). This new model did not have good fit, χ^2^(654) = 1,938.3, RMSEA = 0.099, SRMR = 0.105, and CFI = 0.714, mainly because of bad fit in the personality part of the model. Although the fit was bad, we tested whether the general competence factor correlated with the GFP. The correlation between the general factors was strong at 0.837, *p* < 0.001, suggesting that the competence factor is closely aligned with the GFP^7^.

**FIGURE 2:**
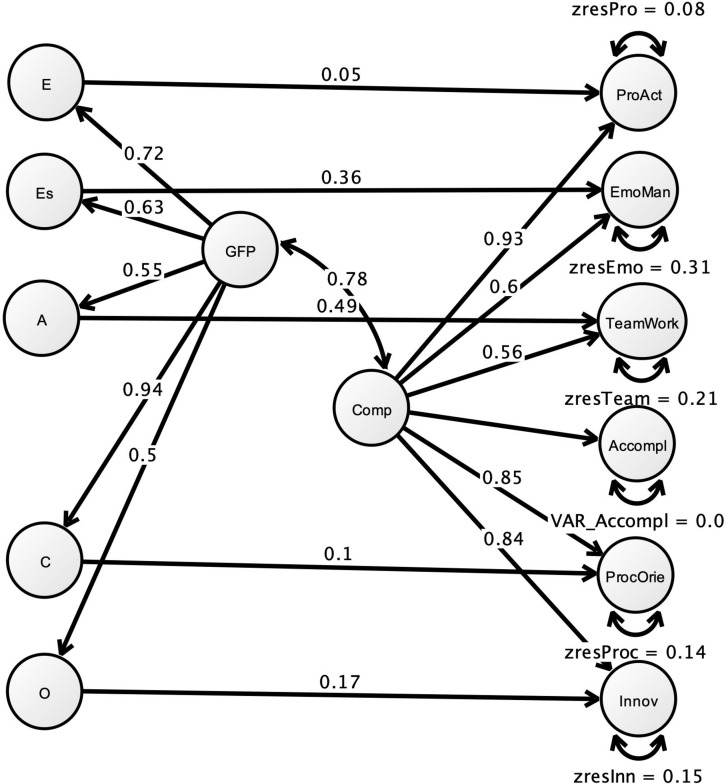
MIMIC model with Big Five variables as covariates for BFC-GRID competence factors and a hierarchical competence factor. E, Extraversion; Es, Emotional stability; A, Agreeableness; C, Conscientiousness; O, Openness; ProAct, Proactive; EmoMan, Emotion management; TeamWork, Teamwork; Accompl, accomplishments; ProcOrie, Process oriented; Innov, Innovative; GFP, General factor of personality; Comp, the higher-order competence factor; zres, unexplained variance in the competence factors. Observed variables not shown.

The main hypothesis was evaluated using the residuals of the competence factors in the merged model. Previously, we showed that the general competence factor explained a large percentage of the specific competence factors, suggesting that all factors were encompassed by the general factor. Before adding the personality factors and the GFP as covariates, the standardized residuals were 0.0 (this was fixed to zero), 0.356, 0.179, 0.098, 0.139, and 0.388, for accomplishment, emotion management, innovative, proactive, process oriented, and teamwork, respectively. The GFP, along with the personality trait covariates for the respective competence factor, was added (the MIMIC model is shown in Figure 2). Three of them were significant: emotional stability to emotional management, β = 0.356, *p* < 0.001, agreeableness to teamwork β = 0.490, *p* < 0.001, and openness to innovativeness β = 0.170, *p* = 0.011. The residuals for these competence factors were reduced to β = 0.309, *p* < 0.001, β = 0.21, *p* < 0.001, and β = 0.148, *p* = 0.006, for emotion management, teamwork, and innovative, respectively. Only teamwork was related to the GFP, β = −0.225 (*p* = 0.008), suggesting a suppressed relation. These results suggest that even if most of the variance in the competence factors can be attributed to a general competence factor, also closely related to the GFP, three factors seem to have specific variance even after controlling for both the general factor and the relevant personality factors. The fit was not good for this model, χ^2^(649) = 1,842.4, RMSEA = 0.096, SRMR = 0.101, and CFI = 0.733. We re-estimated it with the modified personality model, including secondary loadings and correlated error variances. This model had adequate fit, χ^2^(615) = 1,145.9, RMSEA = 0.066, SRMR = 0.070, and CFI = 0.882, and the results in relation to the hypotheses tested above did not differ in any fundamental way.

There are of course a very large number of results presented above, but the most important concern is that competence content tends to create a very general factor in personality inventories and that this factor seems to be so strong that the personality content, as estimated by the Big Five Inventory, was discernible in only three out of five factors.

## Discussion

### Summary of Results

The present research aimed at showing that personality trait models have been dependent on the selection of items used in the measures. We found that, in general, a distinction can be made between what could be called quality and quantity. Some scales and items concerned how well behavior was performed, i.e., the quality, while other items lacked this information and therefore were more related to how often a certain behavior has been performed, i.e., the frequency. Together, the results of the empirical studies reveal that these differences in content are discernable for both the experts and laypersons. The results also suggest rather severe consequences of having this mix in the measures. There were systematic individual differences in how strongly influenced participants were by the mix of competence-related and other content. Lastly, only weak support was found for specific Big-Five competencies. The results will be discussed first in relation to personality theory and then to personality measurement.

### Theory

Personality trait theory is just a subdomain of personality theory, but it is an important domain because it summarizes individual differences between people that can be used to predict behavior. The present research shows that key measures used both to build theory (e.g., Costa and McCrae, 2017) and to predict criteria (Salgado, 1997; Barrick et al., 2003; Poropat, 2009) contain more than just differences in frequencies of behaviors, feelings, and thoughts. Rather, they also include other aspects of individual differences, i.e., of a more qualitative nature. It is obvious from many personality theories, including some trait theories, that ability and adaption have been regarded as one of its main areas of interest. When the Big Five and the FFM were discovered, mainly based on factor analysis, the lexical material and the reanalyzed personality instruments already included a mix of quantity and quality. Because so much of the development of personality trait theory has been built on the analyses of personality inventories, the mix has had a large influence on what has been found in personality research. The discovery of the GFP, for example, is perhaps not that unexpected, given the number of competence-related items in many personality inventories. Personality researchers are often excited by the ability of traits to predict achievements, e.g., in school, academy, or at work, but this is of course not so surprising when the measures assess competence-related matters and not only behavior. For example, if one asks whether someone is goal oriented, it is perhaps rather obvious that this should be related, at least weakly, to academic and work performance.

Two of the studies tested hypotheses about whether competence is global to all factors or specific to each factor of the FFM. The results make it probable that the way we measure the qualitative aspects of competence in personality inventories tends to end up in one single higher-order factor. Previously, both one and two higher-order factors have been suggested to reside above the Big Five. In the present research, there was support for one higher-order factor but not for two higher-order factors^8^.

A legitimate question to ask regarding the finding of a single general factor is whether the GFP advocates, in fact, have discovered a factor that is a consequence of the more or less deliberate inclusion of competence items in personality inventories? At first sight, this could be seen as support for the GFP in general and suggest that individual differences in competence make some people more socially effective than others (van der Linden et al., 2016). However, also non-competence-laden inventories reveal a strong GFP, as suggested by Study 3. In fact, in that study, the competence factor, although larger, had the same substantive content as the GFP from the non-competence version of the inventories. Had there been unique variance, then this would have supported a substantive interpretation of the GFP, but the present studies rather only give a hint at why there is a GFP in many personality inventories.

Another relevant theoretical question is whether ability is associated with the factors of the five-factor structure. For example, DeYoung et al. (2014) claim that cognitive capacity must underlie the cognitive exploration that is the core of Openness to experience, and Tett and Burnett (2003) state that the willingness to help must be accompanied by the capability to help for trait expression to take place. In the present studies, there is only weak support for trait-specific competency. Importantly, the abilities that may underlie trait expression and competence variance specific to latent factors in personality seem to overlap completely or alternatively are not there. In other words, at the level of measurement, individual differences in trait-specific abilities are not distinguishable from the differences in frequency of thoughts, feelings, and behavior related to the Big Five. The abilities that enable trait expression could of course also be asked about separately, in items that do not touch upon frequency of trait expression, which could yield competence variance specific to each FFM dimension. However, our attempt to find strong separate competence factors was unsuccessful in both Studies 3 and 4. Thus, the lack of support for five-factor specific competence variance in the present studies leaves no final answer as to whether competence in Big Five inventories is specific to the different factors.

The question of whether competence should be included in trait theories like the FFM/Big Five is currently completely open. It could be argued that trait theory should only be based on individual differences in the frequency of behaviors, feelings, and thoughts. Or it could be argued that rather than restricting personality traits, the domain covered by traits should be broadened (see for example Saucier, 2019). Many of the examples of what traits can be used for, to predict performance, longevity, health behavior, and so on, may largely rest on the competence aspect of traits more than the frequency of behaviors related to those traits. We presume that it would be hard to let go of that in trait models.

Another way of thinking about traits is that they consist of different components, where one is a quantitative description of how people usually behave, think or feel, and the other, conceptually completely independent component, describes people’s level of competence. Most theories about higher-order factors of traits, e.g., the GFP and de Young’s plasticity and stability factors, seem to conclude that they are related to some competence-related dimension(s) (e.g., social effectiveness). Based on that, a more viable way of thinking about quality is that it is independent of quantity, not necessarily on the measurement level but on the conceptual level (Bäckström and Björklund, 2014).

The results of the present studies and related previous research suggest the possibility that the domain of qualitative aspects consists of a smaller number of factors (one, or maybe two) compared to the quantitative. Interestingly, in adjacent psychological fields, like person perception, there are two factors, agency and communion (Abele and Wojciszke, 2014). Both are typically described as qualitative factors. In a later work, Abele et al. (2016) have suggested that one facet of agency is competence. Some theories of person perception suggest that when people make inferences about traits, they mainly do this based on the dimensions of agency and communion. However, this is still compatible with the notion of individual differences in behavior, thoughts, and feelings as being composed of a completely different set of dimensions, say the Big Five. It appears to be a common denominator of higher-order concepts that they have a qualitative aspect, in the sense of being more clearly related to evaluativeness, adaptivity, and the like, than the personality traits that underlie them. The same may go for test items, such that more concrete personality traits and items are less related to quality than more broad and abstract traits and items are.

### Measurement—What Should We Do About It?

Why are competence-related items included in personality inventories? One likely explanation is the dependence on early lexical analyses. When Allport and others (see review by John et al., 1988) selected adjectives that describe personality differences, they did not exclude adjectives that suggest that the behavior described was generally more competent. Following the historical expose over the lexical approach by John et al. (1988), it seems that Raymond Cattell was struggling with whether to include unipolar traits or not. Others have suggested that there is too little competence-related content in personality. Lately, Morales-Vives et al. (2014) have suggested that concepts like abilities and virtues, both clearly competence related, were ignored by the creators of lexical Big Five models. Maybe, they *should* have been ignored, but the present research shows that there are plenty of them in inventories in use today. Instead, the lexical analyses, which have been the building blocks of personality trait models, have been rather inclusive with regard relation to individual differences in the quality of behavior, e.g., competence and ability. The consequence of this is that almost all facets of personality inventories include competence-related items. Other models based on lexical analysis, such as Wiggins’ circumplex models of interpersonal traits (Wiggins, 1979), also have traits that are more qualitative, the main dimensions being dominance and warmth. It is interesting to note that many very well-cited studies on the lexical hypothesis (Goldberg, 1990; de Raad and Mlačić, 2017) of trait personality do not discuss whether individual differences in frequencies should be complemented with individual differences in quality, e.g., adaptation, competence, and abilities. Still, on the item level, Study 1 shows that items seem to vary regarding the competence/quality dimension.

The results of the present study suggest that raters note that some items include competence and use it to rate themselves as more or less competent. However, it is also important to note that neither the experts nor the naive raters agreed. There were rather large differences between them, but overall, there was rather high agreement. This is akin to how item social desirability is thought to influence ratings. There are many suggestions in the literature to avoid items with an obvious social desirability (e.g., Jackson, 1971). Importantly, an item that is obviously competence related is also obviously desirable. To follow the advice from, for example Jackson, these items should be excluded. On the other hand, many items that are desirable do not refer to competence, for example, “Am often happy,” “Often feel blue,” “Like to visit new places,” “Stick to my chosen path,” and “Love to help others” all have an evaluative tone but do not obviously refer to a competent person.

One could argue that it is inevitable that traits have this evaluative (e.g., competence) component, but it has been shown to be possible to create Big Five inventories that are more evaluatively neutral, without losing criterion validity (Bäckström et al., 2014). One method for reducing social desirability is evaluative neutralization where items are rewritten such that it is not apparent to the rater what would be a socially desirable response (Bäckström et al., 2009). As competence items are generally socially desirable, evaluative neutralization of items in personality inventories would likely reduce the competence factor. This is what we would recommend. Doing the reverse, removing competence-related items to reduce social desirability should not be enough to rid inventories from the evaluative factor, as there is plenty of trait-related content that is unrelated to competence. Another possibility would be to only use items that refer to behaviors, feelings, and thoughts. For example, instead of suggesting that you are organized, they should suggest that you tend to organize things.

To disentangle quantity form quality in personality trait measures is obviously a challenge. All the inventories used in the present study included items that were competence related, and they were distributed over all the factors and facets. Some of the factors (e.g., Conscientiousness) had a larger proportion of competence items than others. But even if competence was unevenly distributed in inventories, the ratings seemed to be influenced in a very general way. The results of Studies 3 and 4 illustrate the difficulties of creating a competence-related inventory that measures more than the general competence factor. This problem resembles that in organizational psychology, when scales have been constructed to measure separate kinds of work performance and it is found that a halo effect influences all the ratings.

The results of the present study suggest that self-ratings of personality include a rather large general competence-related factor, but such a factor stands in stark contrast to the definition of competence within other fields of psychology. Competence, like being convincing, is considered very specific, and to be a competent person does not mean having a general ability to manage a variety of things. This is akin to the debate in social cognitive theory about self-efficacy, where the originator of the concept (Bandura, 1986) rejected attempts to measure general self-efficacy, precisely because self-efficacy is specific to a set of behaviors. Our finding of a general competence-related factor is also relevant to the debate about core self-evaluation (Judge and Bono, 2001), where it is suggested that many important traits, e.g., self-esteem, emotional stability, and general self-efficacy, are influenced by a common factor. Probably, the competence factor in the present research is closely aligned to core self-evaluation.

### Limitations

In the present research, we made a dichotomization between competence items and non-competence items. In future studies, it may be possible to rather estimate the degree to which an item is competence related, which would be useful both when performing statistical analyses (e.g., for estimating linear vs. non-linear models of how competence items influence personality ratings) and selecting items when working with psychometric aspects of personality measurement (enable cutoffs for inclusion, etc.).

Another limitation, in relation to generality, of this research is that we only used a limited number of inventories. Obviously, the selection of inventories was based on our experience with these inventories, e.g., that there was a general factor and that we found competence-related items in them. To be fair, we have only shown that some personality inventories are influenced by unipolar concepts like competence. On the other hand, these inventories are widely used, and at least, the NEO-PI-R has been important to the development of the Big Five/FFM trait theory. In addition, this research is limited to an English-speaking population, but most of the results can probably be applied also in other cultures and languages.

Yet another limitation is that the instruments used to measure specific competences were not very well validated, even if the BFC-GRID has been psychometrically evaluated by the developers of the inventory. We have argued that it is not easy to measure competence specifically for different traits and that the use of competence-related items in many of today’s inventories seem to miss their target. It is possible to argue instead that competence-related content is valuable because they are valid contributors to the traits, but then again, in the present study, they ended up in a global factor.

It may not be possible to separate the specific competence included in each trait with self- or peer ratings, since the difference in frequency always also suggests a difference in competence for the trait. In other words, people who exhibit extraverted behaviors also are competent in delivering this behavior. To test this hypothesis, it would probably be necessary to use personality estimates based on other methods than self-ratings. One obvious reason is that self-ratings tend to result in the very large general factor, akin to social desirability, that probably will obscure a more specific relation between frequency and competence. Peer ratings is a possibility, but also such estimates seem to have a very large common factor (Bäckström and Björklund, 2014). It may be possible to use observational data, e.g., some kind of experience sampling based on both self-observations and peer observations.

It is a limitation of this research that the expert ratings were made by the authors. However, the goal was restricted to demonstrating the existence of a competence factor in ordinary Five Factor instruments, which was the case also when the ratings were made by novices that were unaware of the hypotheses. There were differences between the expert and the naive raters both on a general level (larger number of competence items) and on the rating of specific items. For example, all experts rated “Like to solve complex problems” as non-competent, while 93% of the naive raters rated it as a competence-related item. To us, it was an obvious attitude, but it is easy to understand why the naive raters thought that only competent persons like complex problems. How reliably competence-related items can be separated from other items is a question for future research. Our goal has been to raise the general issue and instigate a much-needed debate about the relationship between trait theory and what is captured by trait measures.

### Future Research

The relative focus on competence in traits has not been systematically studied before within personality theory. Personality trait theory has been almost completely dependent on ratings, and the present research shows some of the limitations of this method. Our results should of course be replicated not only using other samples and other inventories but also using alternative strategies, including other kinds of measures such as peer ratings. Peers may be better at seeing and rating the specific competences of people they know. More objective measures of behavior and competence should also be used, e.g., experience sampling and other more observational techniques. The general question of whether differences in the frequency of behavior is related to competence in the same domain is also interesting. Lastly, the general factor of competence, shown here and in other studies, should be investigated to establish whether some people really are generally more competent in relation to all five factors of the Big Five (or the six of HEXACO). To investigate the relation between trait behavior and trait competence, it would be necessary to conduct an extensive number of studies that disentangle them, such as measuring the number of trait behaviors and to what extent the same behaviors are adaptive in the relevant kind of situation. If it can be shown that adaptation in such cases is unique for a trait, then there would be strong support for the specific trait competence hypothesis.

The present studies have not investigated the competence in the sense of selecting appropriate behaviors in certain situations. It is obvious that the situation has a profound influence on whether it is competent, or not, to perform a certain behavior. Situations are cues for the expression of traits, such that an individual’s competence is related to trait level in this situation, akin to a trait manifestation investigated by Fleeson (2007). Trait activation theory is also relevant; it suggests that traits are latent potentials residing in the individual; therefore, to evaluate the competence aspect of a trait, it is necessary to take situations into account (Tett and Burnett, 2003). On the other hand, to study the relation between the situation, behavior, and competence is much more difficult when the measures of behavior already include references to whether the person is competent or not. For example, whether it is competent to be “good at organizing” in a situation of extreme hurry may not be the right question to ask, rather you should ask whether it is competent or not to engage in a lot of organizing behavior when in a hurry.

## Conclusion

We are not suggesting that competence should be relegated from personality theory. On the contrary, personality is a field within psychology with the ambition to describe and explain whole persons by looking at individual differences. Competence and similar concepts are at the center of the focus in this endeavor. One way to react to the present results is that competence in personality inventories is not much of a problem, since it may actually be beneficial in, e.g., a recruitment and selection context (McCrae and Costa, 1983; Paunonen and LeBel, 2012) for a related discussion on the benefits of social desirability in personality measures. It is true that including competence in personality measures may have benefits in some contexts. However, there are drawbacks, too. For example, when the focus is not on predicting behavior but identifying personality differences in a broader fashion, competence-free inventories should provide a higher level of differentiation. Judging from the present results, it appears that competence-related items add a general fuzziness to personality measures, making the facets correlate since referral to competence influences all ratings in the same way. Thus, the question is rather whether it is a good idea to mix the frequency of trait behaviors with the level of the quality of such behaviors in measures of personality traits.

## Data Availability Statement

The original contributions presented in the study are publicly available. This data can be found here: https://www.openicpsr.org/openicpsr/project/123081/version/V1/view.

## Ethics Statement

The studies involving human participants were reviewed and approved by the Swedish ethical review authority. The patients/participants provided their written informed consent to participate in this study.

## Author Contributions

MB made the main part of data analyses and preparation. All authors contributed to study conceptualization, data collection, and report writing.

## Conflict of Interest

The authors declare that the research was conducted in the absence of any commercial or financial relationships that could be construed as a potential conflict of interest.

## Footnotes

1 A few participants reported a somewhat higher age.

2 Data and scripts for the statistical analyses can be found at: https://www.openicpsr.org/openicpsr/project/123081/version/V1/view

3 In total, they provided responses to about 300 items, the rest were not used in this study. The same material has been used in another study that dealt with a different research question. We excluded 51 participants with unreliable responses based on very short response times. In all other studies, we included warnings to not respond carelessly and used all participants.

4 www.pimahb.se

5 We also tested a model based on alpha and beta (Digman, 1997). This model, based on four indicators per inventory revealed worse fit than the one general factor model, e.g., ΔRMSEA = 0.001, ΔBIC = 12.2.

6 MIMIC stands for multiple indicators, multiple causes with covariates.

7 We also tested a model based on alpha and beta (Digman, 1997), but that model resulted in similar or worse fit than the one general factor model, e.g., ΔRMSEA = 0.000, ΔBIC = 20.3.

8 We did not present these results more than in footnotes since it would distract from our main results, but it may be interesting anyway.
